# Supplementary material for: Paradoxical Role of AT-rich Interactive Domain 1A in Restraining Pancreatic Carcinogenesis
Source: Cancers (Basel). 2020 Sep 21;12(9):2695. doi: 10.3390/cancers12092695 (PMC7564752; doi:10.3390/cancers12092695)
Supplement: Supplementary file 1 [file cancers-12-02695-s001.zip › cancers-908833-supplemental-final/Cancers-908833 - supplementary .pdf]

Supplementary Materials

Paradoxical Role of AT-rich Interactive Domain 1A in Restraining Pancreatic Carcinogenesis

Sammy Ferri-Borgogno, Sugata Barui, Amberly M McGee, Tamara Griffiths, Pankaj K Singh, Cortt G Piett, Bidyut Ghosh, Sanchari Bhattacharyya, Aatur Singhi, Kith Pradhan, Amit Verma, Zac Nagel, Anirban Maitra and Sonal Gupta

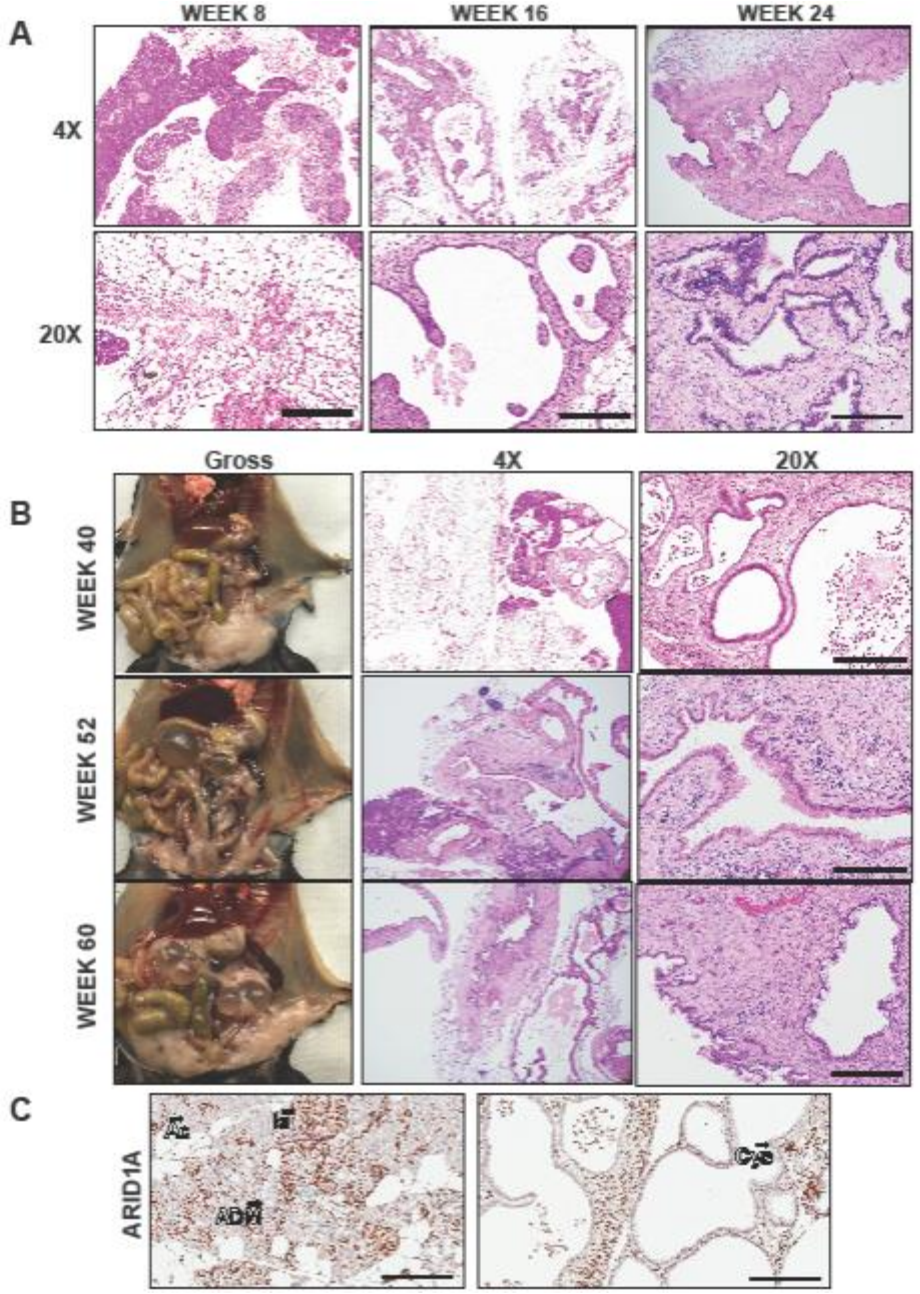

Figure S1. Loss of Arid1a in murine pancreas leads to loss of epithelial homeostasis. Owing to its critical role in embryonic development [1], we conditionally deleted *Arid1a* in the pancreatic epithelial compartment by crossing *Ptf1a-Cre* [2] and *Arid1a*$^{fl/fl}$ mice [1] to derive *Arid1a*$^{fl/fl}$; *Ptf1a-Cre* ("AC") mice. These mice were necropsied at regular intervals and pancreas fixed in formalin for histological assessment. (**A**) Representative microscopic images of H&E-stained pancreatic sections from "AC" mice at indicated ages revealed widespread parenchymal atrophy accompanied by inflammation

starting at 8-weeks, with progressive fatty replacement of normal parenchyma, dilated ducts and microscopic cysts lined by mucinous epithelium at 16- and 24-weeks. *Upper panel*, low magnification at 4× objective lens, *Bottom panel*, high magnification at 20× objective lens. (**B**) Gross images of necropsied "AC" mice at indicated older ages (*left panel*) revealed fluid-filled macroscopic cysts, while representative microscopic images of H&E-stained pancreatic sections at low magnification (*middle panels*) and high magnification (*right panels*) revealed complete loss of normal parenchyma accompanied by large dilated mucinous cystic ducts and few LG-PanINs. (**C**) Representative microscopic images of IHC on pancreatic sections from 40-wk old "AC" mice confirmed lack of ARID1A expression in Ac: acini; Is: islet; ADM (*left panel*) and Cys: cysts (*right panel*) Scale bar is 100 μm.

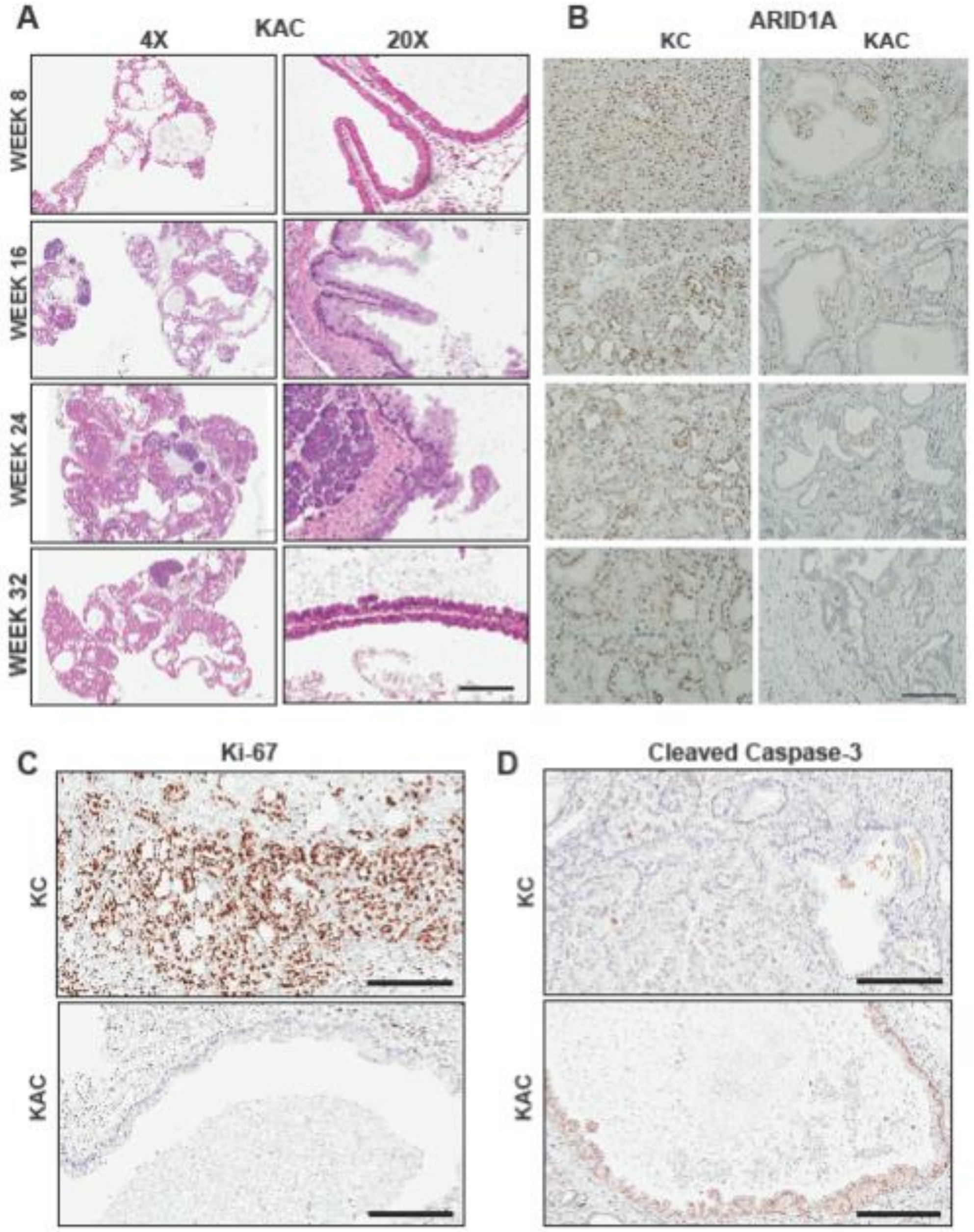

Figure S2. Immunohistochemical characterization of "KAC" pancreas. (**A**) Representative microscopic images of H&E-stained pancreatic sections from "KAC" mice at indicated ages revealed vast network of mucinous cysts resembling low-grade branched duct gastric type IPMN (LG-IPMN) in humans, admixed with ADM and LG-PanINs. (**B**) Representative microscopic images of IHC for ARID1a in pancreatic sections from "KC" and "KAC" mice at indicated ages, confirmed complete lack of Arid1a expression in "KAC", as expected from a *Ptf1a-Cre* driver line. Representative high-

magnification images of IHC staining on pancreata from “KC” (*upper panels*) and “KAC” (*lower panels*) mice for Ki67 (**C**) and cleaved caspase-3 (**D**). Scale bar is 100 μm.

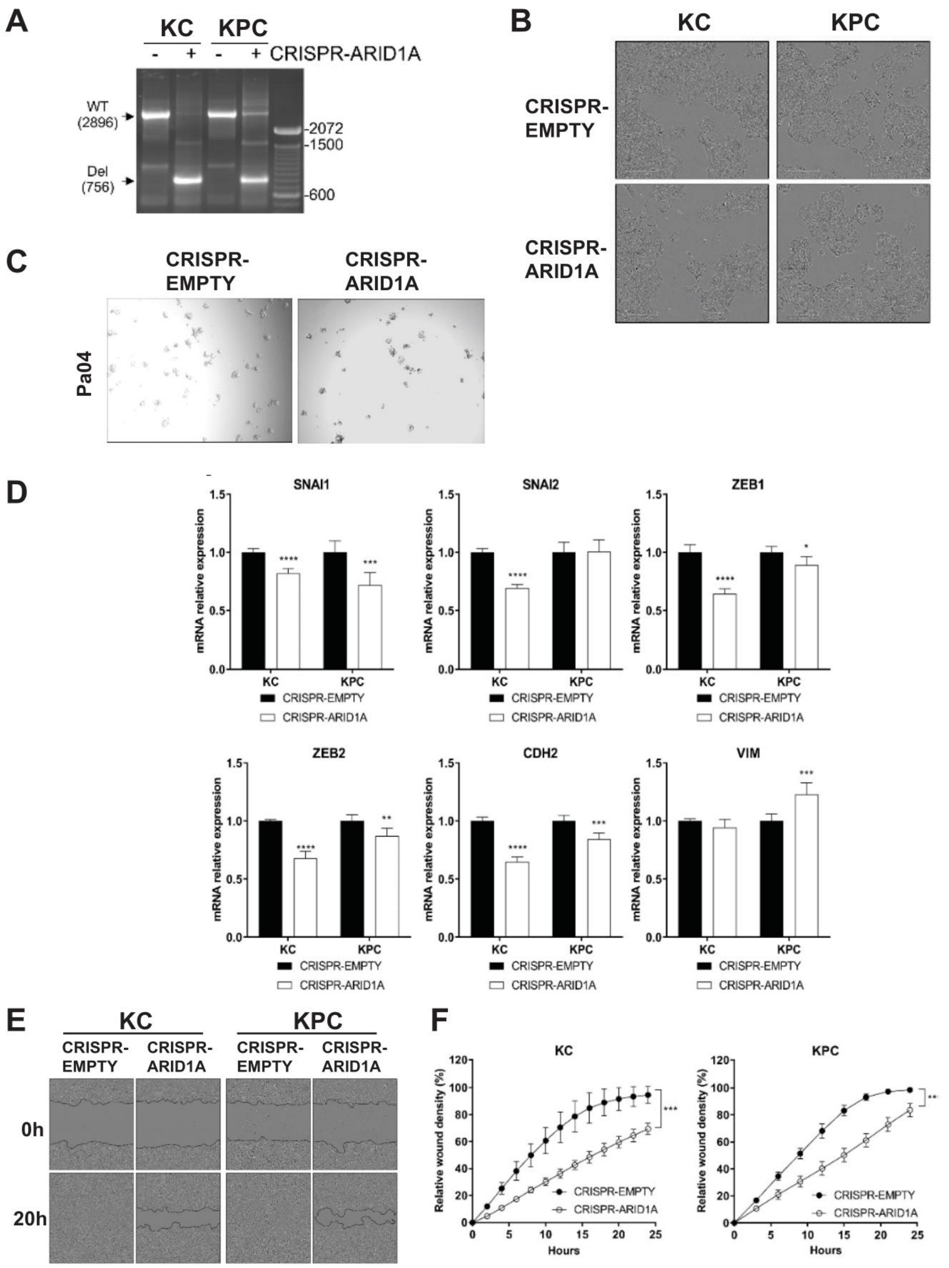

Figure S3. Characterization of *Arid1a*-deleted “KC” and “KPC” cell lines using CRISPR-Cas9 system. (**A**) Genomic PCR on DNA isolated from puromycin-selected “KC” and “KPC” cell lines transfected with CRISPR/Cas9 plasmids, detected wild-type (2896 bp) and deleted (756 bp) ARID1A bands. (**B**) Microscopic images showing cellular morphology of *Arid1a*-deleted “KC” and “KPC” cell lines. Scale bars, 100 μm. (**C**) Representative images of *Arid1a*-deleted Pa04 isogenic cell lines colony growth on soft agar. (**D**) Semi-quantitative RT^2 PCR for various EMT-associated genes on RNA extracted from *Arid1a*-deleted “KC” and “KPC” isogenic cell lines. Values are expressed as relative expression compared to CRISPR-EMPTY for each cell line. (**E**) Representative microscopic images showing migration of *Arid1a*-deleted “KC” and “KPC” cell lines, using Incucyte ZOOM real-time tracking assay. Cells grown to confluence were scratched using the Incucyte Wound maker tool and wound closure tracked every 2 hours post-scratch wound. (**F**) Quantification of wound density data represented as mean ± SD. Results shown are representative from three independent experiments. *, $p < 0.05$; **, $p < 0.01$; ***, $p < 0.001$; ****, $p < 0.0001$.

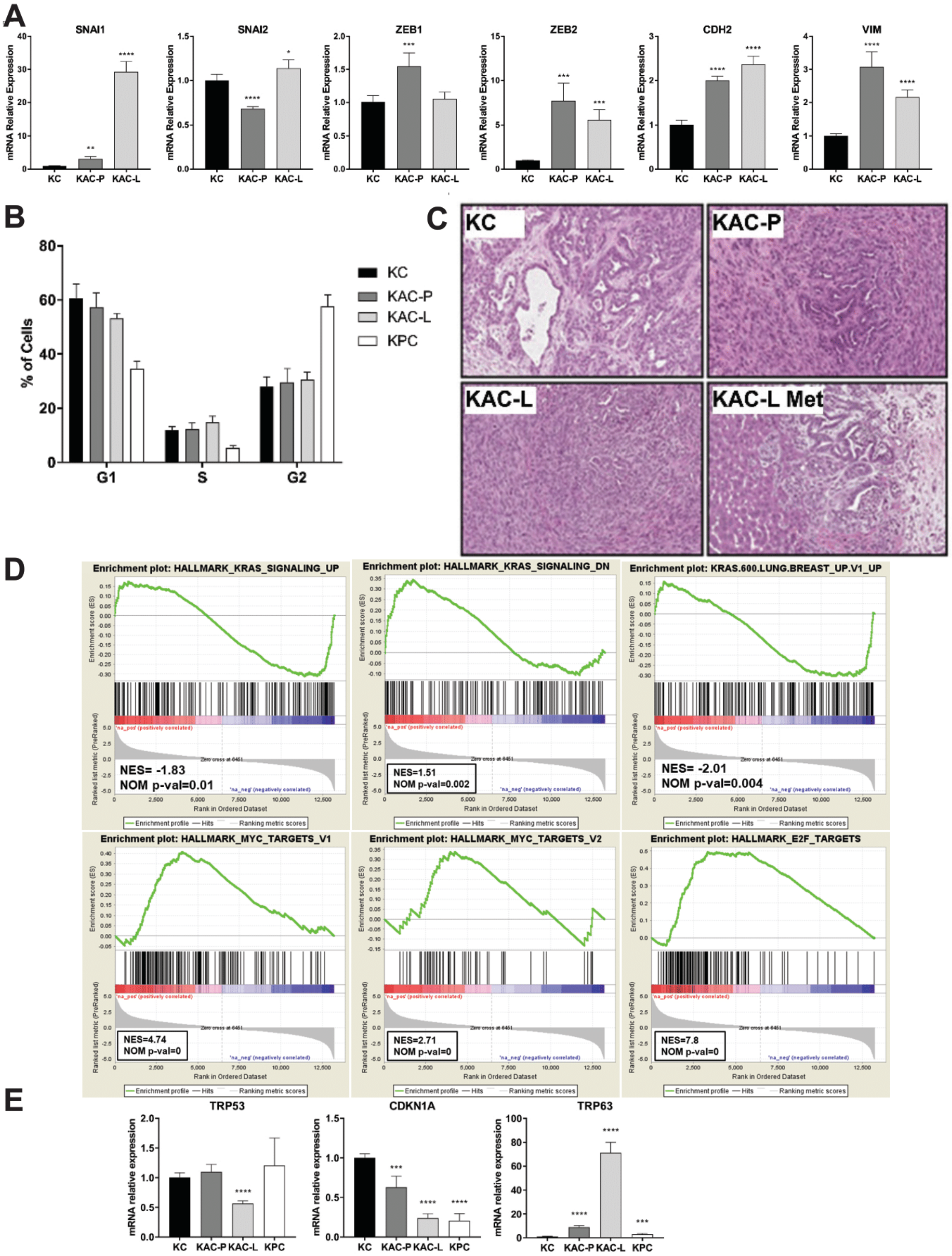

Figure S4. Characterization of autochthonous mouse *Arid1a*-null PDAC cell lines. (**A**) Semi-quantitative RT² PCR for various EMT-associated genes on RNA extracted from "KC" and "KAC" cell lines revealed higher expression of these genes. Ct values were normalized to Gusb and fold change expression was relative to "KC" group. (**B**) Propidium iodide staining was done on cultured cell lines followed by flow cytometric analyses to assess DNA content in various phases of cell cycle. Histogram indicates the percentage of cell lines in each phase of the cell cycle. (**C**) Representative microscopic images of H&E-stained sections of mouse pancreatic tumors harvested 4 weeks after orthotopically implanted with "KC", "KAC-P" and "KAC-L" cells in the pancreas of athymic nude mice (n = 7 mice per group). "KAC-L" cells also metastasized to liver (KAC-L Met). Scale bar is 100 µm. (**D**) Gene set enrichment analysis (GSEA) of differentially expressed transcripts in RNA-Seq of "KC" and "KAC" cells showed enrichment in gene sets associated with downregulated Kras signaling (*upper panel*) and positive enrichment in hallmark gene signature for Myc and E2F targets (*lower panel*).

(**E**) Semi-quantitative RT² PCR showing reduced expression of direct p53 target gene CDKN1A and increased expression of Trp63 in "KAC" cell lines, relative to "KC". "KPC" cells with mutant p53 also showed reduced CDKN1A expression, as expected. Ct values were normalized to Actin and fold change expression was relative to "KC" group. Representative findings from at least 3 independent experiments are shown and data analyzed using the two-tailed unpaired Student's *t* test and considered significant if *, $p < 0.05$; **, $p < 0.01$; ***, $p < 0.001$; ****, $p < 0.0001$, unless otherwise specified.

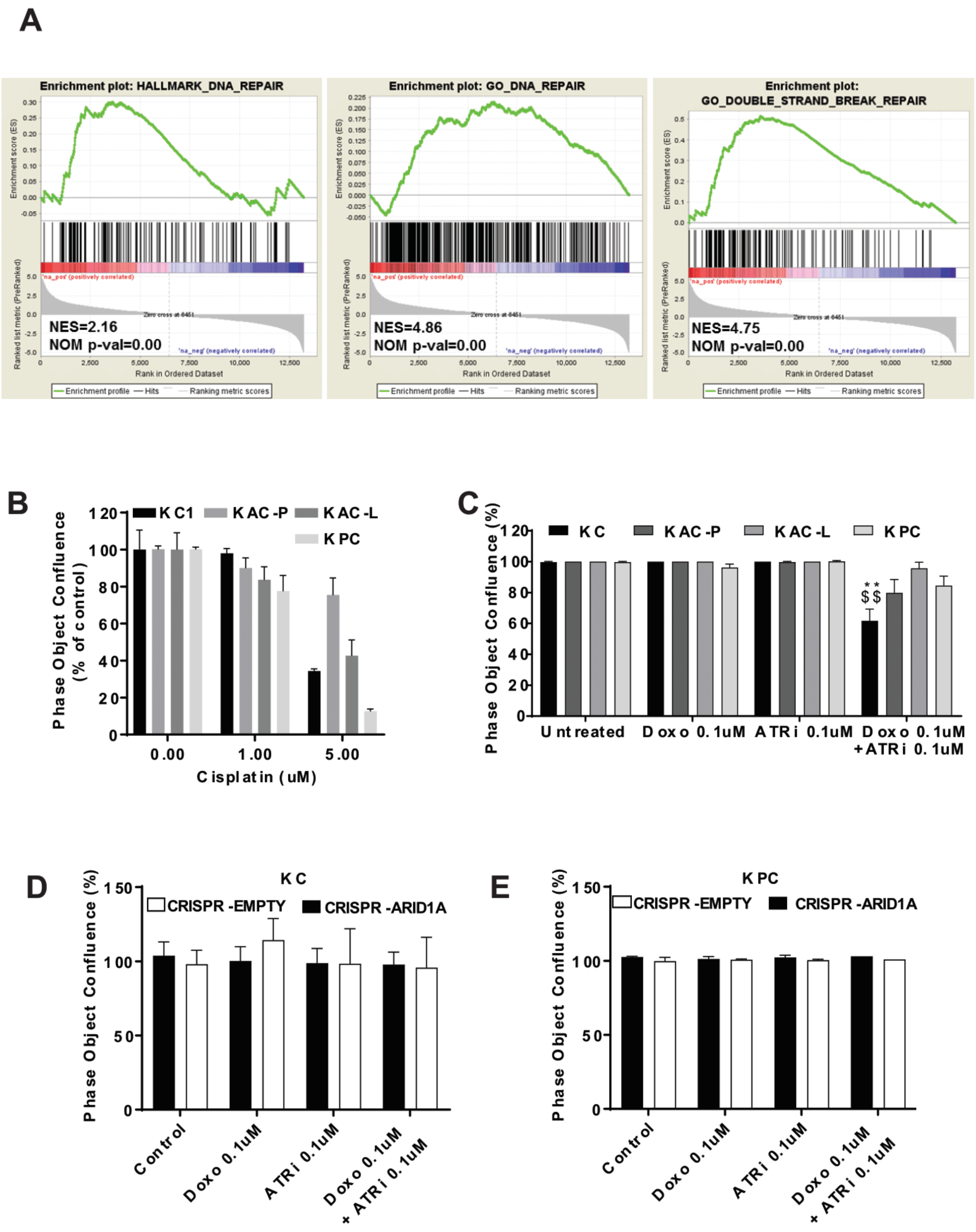

Figure S5. PDAC cells from *Arid1a*-null "KAC" mice shows enhanced DNA repair capability. (**A**) GSEA analysis of differentially expressed transcripts in RNA-Seq of "KC" and "KAC" cells showed enrichment in signatures associated with DNA repair in "KAC" cells compared to KC control. (**B**) Monolayer culture of autochthonous PDAC cell lines were treated either with increasing doses of Cisplatin (B) or combination of Doxorubicin and ATRi (**C**) for 72 h. Cell confluency was imaged and quantified by Incucyte ZOOM and data represented as % of the corresponding vehicle control for

each cell line. ****, $p < 0.0001$ for 5 uM Cisplatin. (**D–E**) Sensitivity of *Arid1a*-deleted isogenic "KC" (D) and "KPC" (E) cell lines to 72 h of Doxorubicin and ATRi treatment, evaluated as in (B). Representative findings from at least 3 independent experiments are shown and data analyzed using the two-tailed unpaired Student's *t* test. **, $p < 0.01$ when compared to Doxo alone; $^{\$\$}$, $p < 0.01$ when compared to ATRi alone.

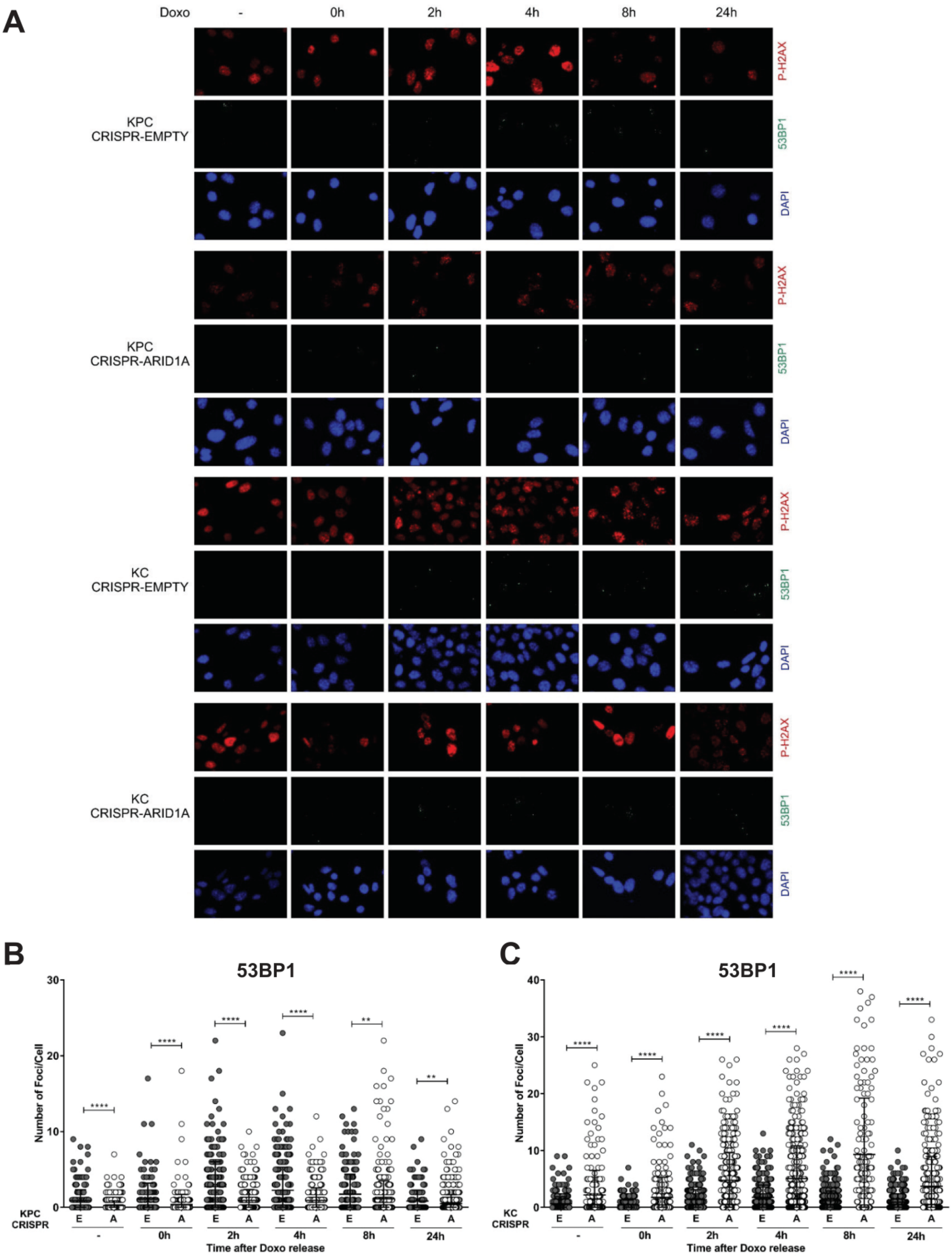

Figure S6. Assessment of DNA damage post *Arid1a*-deletion in PDAC cells. (**A**) Immunofluorescence staining for P-H2AX, 53BP1 and DAPI showing nuclear foci after Doxo treatment in *Arid1a*-deleted isogenic "KC" and "KPC" cell lines. Cells were exposed to 0.1 uM Doxo for 30 min and fixed at indicated timepoints. Representative images shown from at least 3 independent experiments. Scale bar, 20 μm. (**B–C**) Scatter plots showing quantification of the number of 53BP1 foci/cell in "KPC" (left) or "KC" (right) isogenic cell lines, performed with iMaris Microscopy Image Analysis Software

(Bitplane). Data are represented as mean ± SD and two-tailed unpaired Student's *t* test have been used for data analysis (unless otherwise indicated) and considered significant if *, $p < 0.05$; **, $p < 0.01$; ***, $p < 0.001$; ****, $p < 0.0001$, unless otherwise specified.

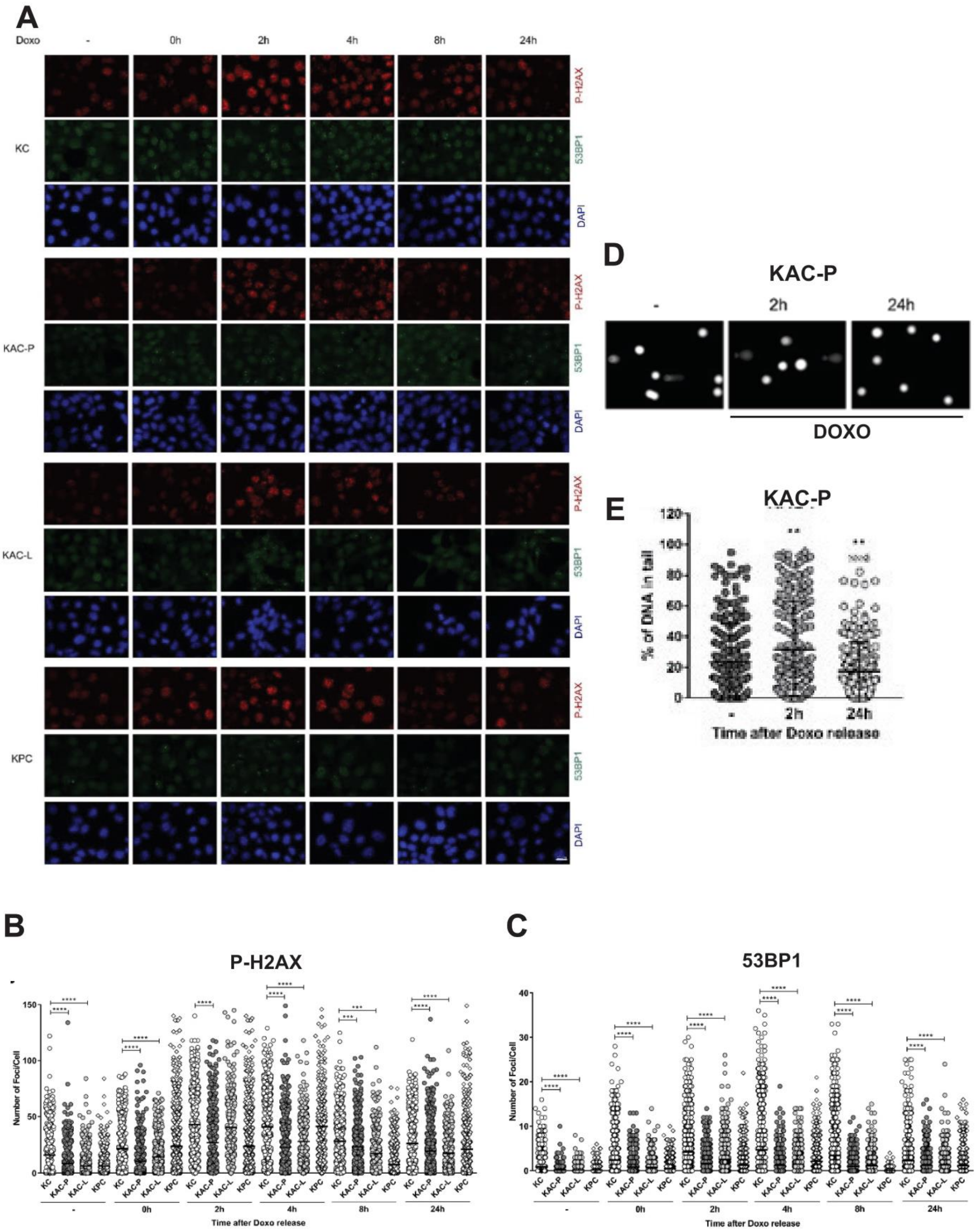

Figure S7. Assessment of DNA damage repair in autochthonous mouse PDAC cell lines. (**A**) Immunofluorescence staining for P-H2AX, 53BP1 and DAPI showing nuclear foci after Doxo treatment in autochthonous *Arid1a*-null cell lines. Cells were exposed to 0.1 uM Doxo for 30 min and fixed at indicated timepoints. Representative images shown from at least 3 independent experiments. Scale bar, 20 µm. (**B**–**C**) Scatter plots showing quantification of the number of P-H2AX (B) or 53BP1 (C) foci/cell, performed with iMaris Microscopy Image Analysis Software (Bitplane). (**D**–**E**) Representative images from Comet assay performed after exposing KAC-P cells to 0.1 uM Doxo for 30 min and then released for 2 or 24 h. Quantification indicated as mean ± SD with two-tailed unpaired Student's *t* test used for data analysis; *, $p < 0.05$; **, $p < 0.01$; ***, $p < 0.001$; ****, $p < 0.0001$. *two-tailed

unpaired Student's *t* test against untreated sample, $two-tailed unpaired Student's *t* test against 2 h timepoint.

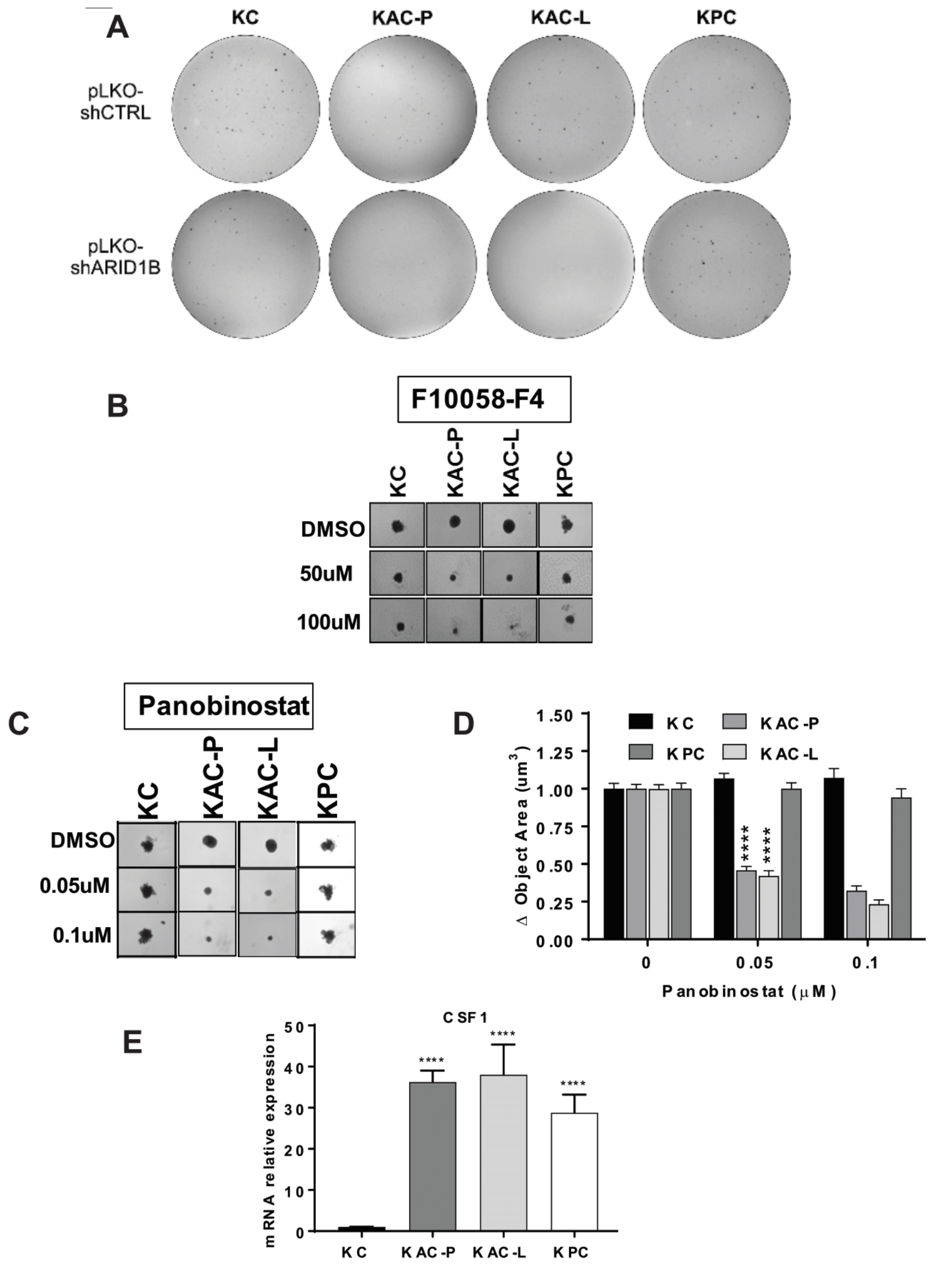

Figure S8. Synthetic lethality in autochthonous *Arid1a*-null PDAC cells. (**A**) Representative well images of colonies in soft agar, with 14 d of growth post transduction with pLKO-shARID1B vector. (**B**) Representative images of F10058-4 treated spheroids from autochthonous cell lines grown on ultralow attachment plates for 7 d. (**C**–**D**) Representative images of Panobinostat treated spheroids from autochthonous cell lines grown on ultralow attachment plates for 7 d. Images were captured and spheroid Area (um3) measured using spheroid imaging protocol of the Gen5 Image software on Cytation 3 (Biotek) using 10× objective lens; data normalized to vehicle control and plotted as change relative to control. **E,** Relative expression levels of Csf1 assessed by semi-quantitative RT^2 PCR in "KAC" cell lines. Ct values were normalized to Actin and fold change expression was relative to "KC" group. Two-tailed unpaired Student's *t* test have been used for data analysis and considered significant if *, $p < 0.05$; **, $p < 0.01$; ***, $p < 0.001$; ****, $p < 0.0001$.

Figure 3A

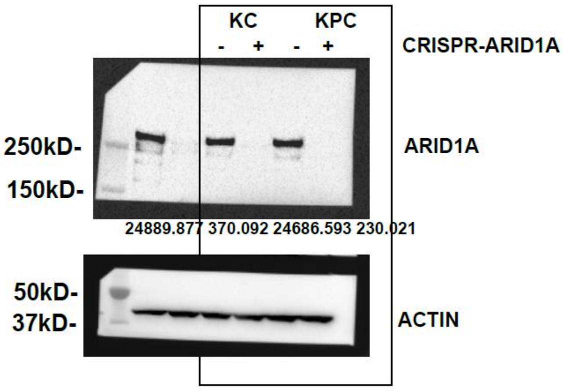

ARID1A	24889.88	370.092	24686.59	230.021
ACTIN	9033.56	7555.246	9495.974	9749.731
Normalized on actin	2.755268	0.048985	2.59969	0.023593
Ratio to Control	1	0.017779	1	0.009075

Figure 3H

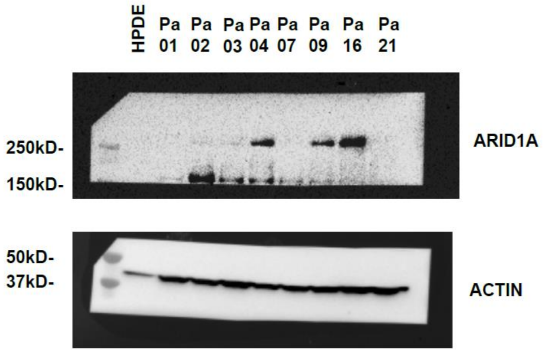

ARID1A	593.506	640.577	2072.397	3193.004	21524.67	137.556	19519.4	43015.09	1308.912
ACTIN	13249.28	33105.64	32985.39	37006.46	28086.74	24462.64	33788.61	33934.69	30183.84
Normalized on actin	0.044795	0.019349	0.062828	0.086282	0.766364	0.005623	0.577692	1.267584	0.043365
Ratio	1	0.431953	1.40255	1.926145	17.10813	0.125529	12.89624	28.29724	0.968062

Figure 3I

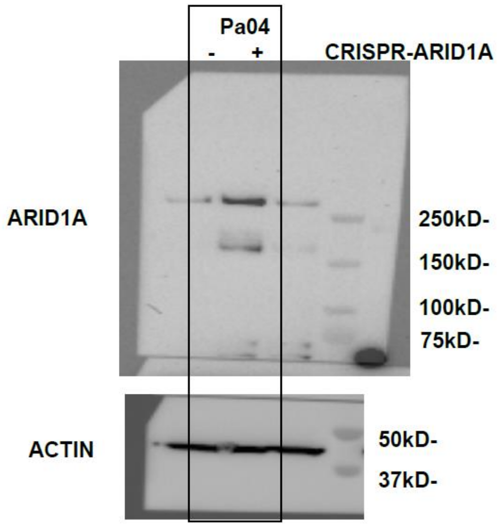

Normalized on actin	1.842455	0.218482
Ratio	1	0.118582

Figure S9. Detail information about Figure 3.

Figure 4E

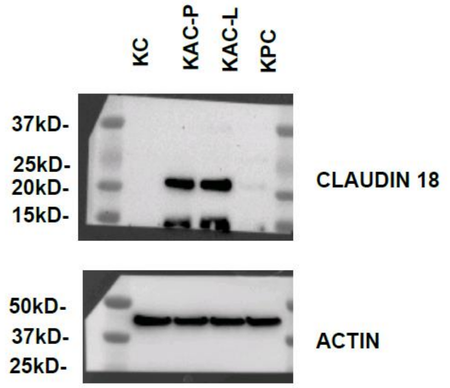

CLAUDIN 18	879	40421.61	44810.56	1079.92
ACTIN	49302.3	45002.6	45833.65	40344.68
Normalized on actin	0.017829	0.898206	0.977678	0.026767
Ratio	1	50.37955	54.83708	1.501356

Figure S10. Detail information about Figure 4.

Figure 5C

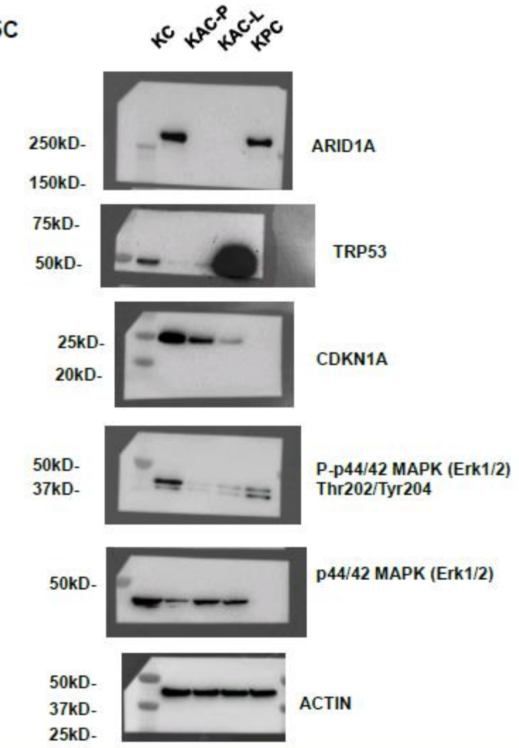

	ARID1A	43843.02	16.121	649.678	32479.79
	TRP53	10818.26	702.335	3814.853	102356.5
	CDKN1A	37543.22	21974.08	5481.146	166.778
	P-p44/42	30427.05	1504.477	4694.61	20171.44
	p44/42	37818.92	15318.23	22858.1	19908.86
	ACTIN	49302.3	45002.6	45833.65	40344.68
ARID1A	Normalized on actin	0.889269	0.000358	0.014175	0.805058
	Ratio	1	0.000403	0.01594	0.905302
TRP53	Normalized on actin	0.219427	0.015607	0.083233	2.537051
	Ratio	1	0.071124	0.379318	11.56216
CDKN1A	Normalized on actin	0.99271	1.434505	0.23979	0.008377
	Ratio	1	1.445039	0.241551	0.008439
P-p44/42	Normalized on actin	0.617153	0.033431	0.102427	0.499978
	Ratio	1	0.05417	0.165967	0.810136
p44/42	Normalized on actin	0.767082	0.340385	0.498719	0.493469
	Ratio	1	0.443741	0.65015	0.643307

Figure 5C

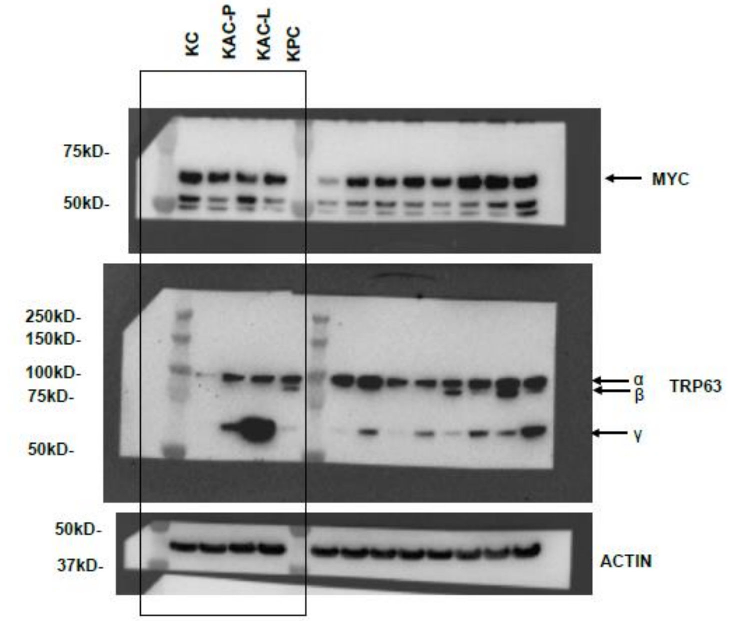

	MYC	203278.9	196486.7	197109.5	197285.1
	TRP63 alpha	5016.167	14082.7	14797.12	10167.36
	TRP63 beta	1771.698	8218.368	11022.63	28470.52
	TRP63 gamma	788.021	30395.04	93396.25	2597.134
	ACTIN	7552.619	7614.518	8395.64	8940.761
MYC	Normalized on actin	26.91501	25.80423	23.4776	22.06581
	Ratio	1	0.95873	0.872286	0.819833
TRP63 alpha	Normalized on actin	0.664163	1.849454	1.762476	1.137192
	Ratio	1	2.784641	2.653682	1.712219
TRP63 beta	Normalized on actin	2.248288	0.270385	0.11802	10.96228
	Ratio	1	0.120263	0.052493	4.875837
TRP63 gamma	Normalized on actin	0.104337	3.991722	11.12437	0.290482
	Ratio	1	38.25781	106.6192	2.784067

Figure 5F

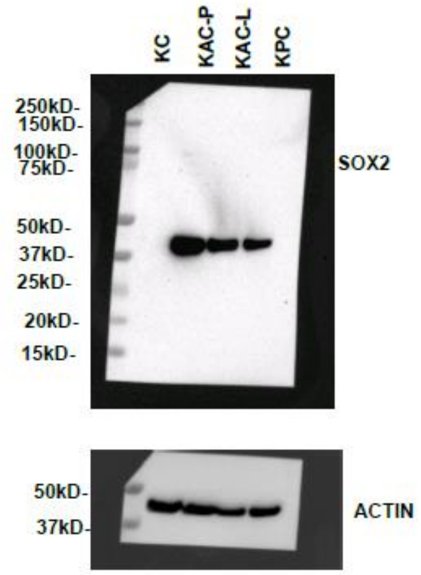

SOX2	2308.749	79657.76	45432.36	35635.88
ACTIN	56705.48	58946.25	43672.55	44370.33
Normalized on actin	0.040715	1.351363	1.040296	0.803146
Ratio	1	33.19099	25.55083	19.72618

Figure S11. Detail information about Figure 5.

Figure 6G

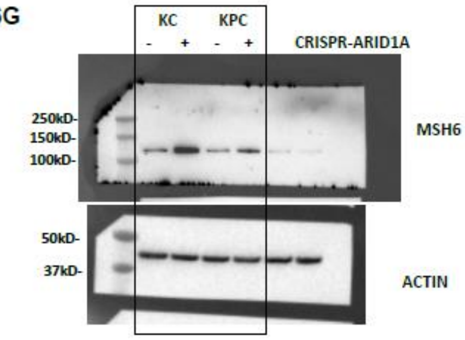

MSH6	17410.49	50076.67	11679.57	21238.08
ACTIN	38656.75	39388.21	37172.02	33890.61
Normalized on actin	0.450387	1.271362	0.314203	0.626666
Ratio	1	2.822822	1	1.994459

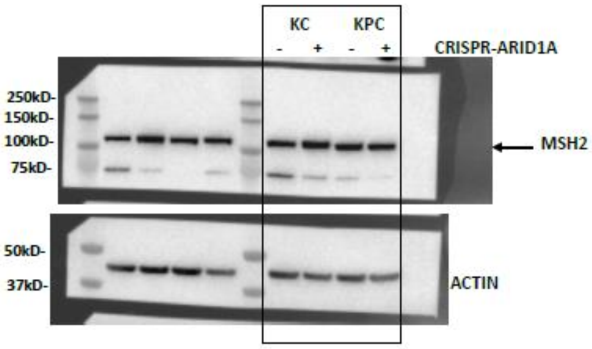

MSH2	45588.23	55386.25	52140.3	44798.58
ACTIN	46884.94	38690.72	41744.01	35999.33
Normalized on actin	0.972343	1.431513	1.249049	1.244428
Ratio	1	1.47223	1	0.996301

Figure 6G

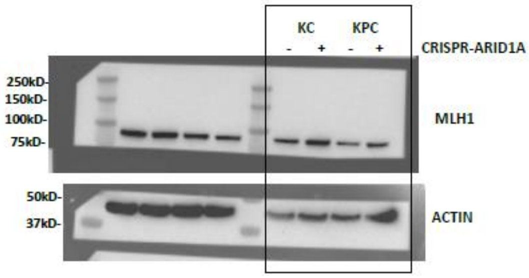

MLH1	25561.91	37374.75	18348.96	22586.22
ACTIN	26659.51	41814.6	38089.7	57160.91
Normalized on actin	0.958829	0.893821	0.48173	0.395134
Ratio	1	0.9322	1	0.820239

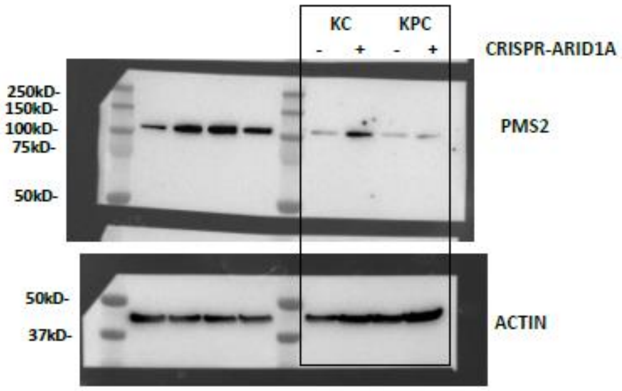

PMS2	5628.631	24632.71	4067.296	5923.974
ACTIN	28368.9	44502.92	45302.45	49895.45
Normalized on actin	0.198409	0.553508	0.089781	0.118728
Ratio	1	2.789738	1	1.322416

Figure 6I

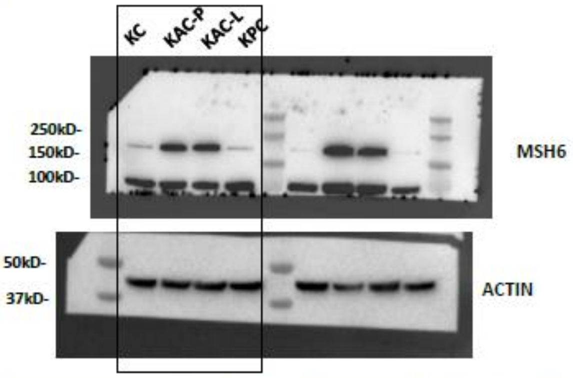

MSH6	6909.459	46509.79	46486.74	5409.752
ACTIN	47457.86	48433.54	48686.17	50325.67
Normalized on actin	0.145591	0.960281	0.954824	0.107495
Ratio	1	6.595721	6.558244	0.738332

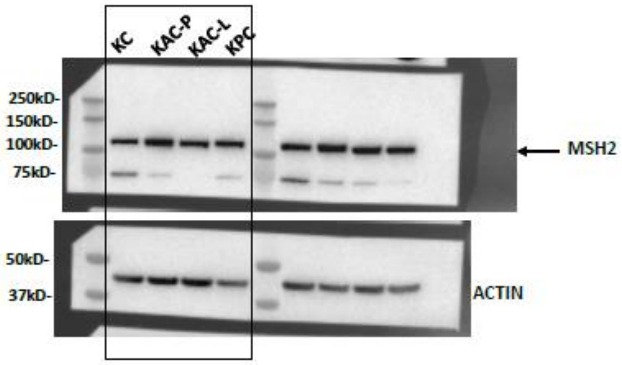

MSH2	31345.98	51850.48	43249.33	41857.63
ACTIN	30049.43	39220.83	35932.31	22543.61
Normalized on actin	1.043147	1.322014	1.203633	1.856741
Ratio	1	1.267332	1.153848	1.779941

Figure 6I

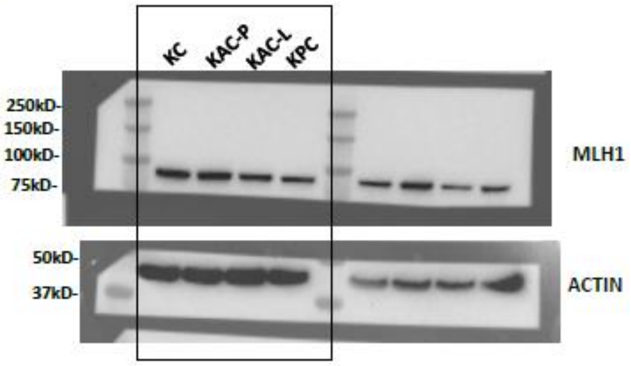

MLH1	38736.58	38871.26	29686.51	23192.56
ACTIN	59829.7	67063.32	66280.35	53598.99
Normalized on actin	0.647447	0.57962	0.447893	0.432705
Ratio	1	0.895239	0.691783	0.668325

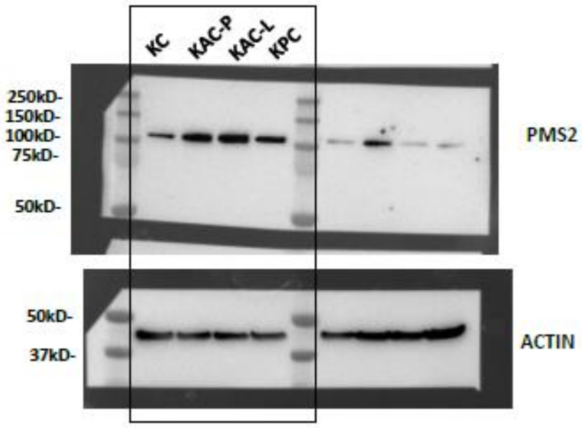

PMS2	17564.23	46209.33	51853.43	36071.69
ACTIN	39982.11	31496.65	35027.89	29103.99
Normalized on actin	0.439302	1.467119	1.480347	1.239407
Ratio	1	3.339658	3.369769	2.821308

Figure S12. Detail information about Figure 6.

Figure 7C

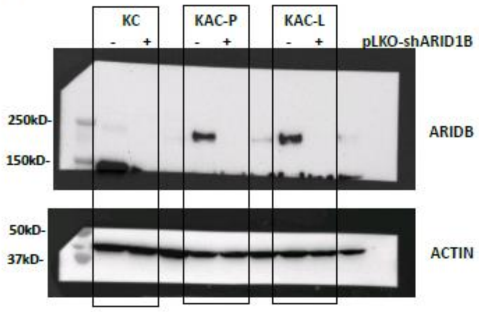

ARID1B	2914.062	91.364	51959.98	173.364	62535.14	125.95
ACTIN	37877.05	45222.48	31485.24	32034.75	36976.35	33695.04
Normalized on actin	0.076935	0.00202	1.650296	0.005412	1.69122	0.003738
Ratio	1	0.02626	1	0.003279	1	0.00221

Figure 7D

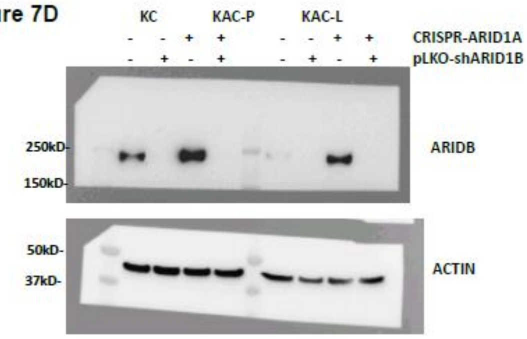

ARID1B	42654.66	1344.092	74774.85	2406.406	4229.134	317.192	48776.81	489.021
ACTIN	40047.16	41810.02	41091.78	40120.61	31548.73	18691.56	21732.58	25514.07
Normalized on actin	1.065111	0.032148	1.819704	0.059979	0.134051	0.01697	2.244409	0.019167
Ratio	1	0.030182	1	0.032961	1	0.126592	1	0.00854

Figure S13. Detail information about Figure 7.

Table S1. Immunohistochemical staining for ARID1A on human IPMNs classified based on dysplasia grade and subtypes.

Sample#	ARID1A Expression	Dysplasia Grade	IPMN Subtype
IPMN-1	-	Low	Gastric
IPMN-2	-	Low	Gastric
IPMN-3	-	Low	Gastric
IPMN-4	-	Low	Gastric
IPMN-5	-	Low	Gastric
IPMN-6	-	Low	Gastric
IPMN-7	-	Low	Gastric
IPMN-8	-	Low	Gastric
IPMN-9*	-	Low	Gastric
IPMN-9*	+++	High	Gastric
IPMN-10*	-	Low	Gastric
IPMN-10*	+++	High	Gastric
IPMN-11	+	Low	Gastric
IPMN-12	+	Low	Pancreatobiliary
IPMN-13	+	Low	Gastric
IPMN-14	+	Low	Gastric
IPMN-15	+	Low	Gastric
IPMN-16	+	Low	Gastric
IPMN-17	+	Low	Gastric
IPMN-18	+	Low	Gastric
IPMN-19	+	Low	Gastric
IPMN-20	+	Low	Gastric
IPMN-21	+	Low	Pancreatobiliary
IPMN-22	+	Low	Gastric
IPMN-23	+	Low	Gastric
IPMN-24	+	Low	Gastric
IPMN-25	+	Low	Gastric
IPMN-26	+	Low	Gastric
IPMN-27	+	Low	Gastric
IPMN-28	+	Low	Gastric
IPMN-29	+	Mix	Pancreatobiliary
IPMN-30	+	Mix	Pancreatobiliary
IPMN-31	+	Mix	Pancreatobiliary
IPMN-32	+	Mix	Gastric
IPMN-33	+	Mix	Gastric
IPMN-34	+	Mix	Intestinal
IPMN-35	+	High	Intestinal
IPMN-36	+	High	Gastric
IPMN-37	+	High	Gastric
IPMN-38	+	High	Pancreatobiliary

IPMN-39	+	High	Pancreatobiliary
IPMN-40	+	High	Pancreatobiliary
IPMN-41	+	High	Gastric
IPMN-42	+	High	Gastric
IPMN-43	+	High	Pancreatobiliary
IPMN-44	+	High	Gastric
IPMN-45	+	High	Intestinal
IPMN-46	+	High	Intestinal
IPMN-47	+	High	Intestinal
IPMN-48	+	High	Gastric
IPMN-49	++	High	Pancreatobiliary
IPMN-50	++	High	Pancreatobiliary
IPMN-51	++	High	Pancreatobiliary
IPMN-52	++	High	Intestinal
IPMN-53	++	High	Intestinal

*Cases with regions of both low and high grade dysplasia.

Supplementary Material and Methods

Genetically Engineered Mice

All mice experiments were approved by UT MD Anderson Institutional Animal Care and Use Committee (IACUC)(code:#00001222-RN02) and performed in accordance with the NIH guidelines(https://grants. nih. gov/grants/olaw/guide-for-the-care-and-use-of-laboratory- animals. pdf) for use and care of live animals under the protocol number 00001222-RN01. *LSL-KrasG12D* [3], *Ptf1a-Cre*/+ [2], *ARID1A$^{fl/fl}$* [1] mice have been described before and were purchased from Jackson laboratories (Item code: Stock No: 023329. Jackson Laboratories, Bar Harbor, Maine, USA). Genotyping PCR was performed from genomic DNA from tails using standard protocol of N-Extract kit (Sigma).

Orthotopic Implantation

Orthotopic tumors were established in athymic nude mice, as per established protocol (47). Briefly, cells were suspended in ice-cold PBS and Matrigel in a 1:1 ratio, and injected into the pancreatic parenchyma of anesthetized mice.

Viral Transduction

Lentiviral infections were performed using 293LTV cells (Cell Biolabs, Cat#LTV-100) as producers of viral supernatants, upon co-transfection with MISSION shRNA lentiviral DNA (Sigma-Aldrich) and the helper vectors pCMVR8.74 (Addgene Cat#22036) and pCMV-VSV-G (Addgene, Cat #8454) using Lipofectamine 3000 transfection reagent (ThermoFisher Scientific, Cat# L3000008). Supernatants from 293LTV cultures was passed through a 0.45 μm filter before transduction of cancer cells, with successful clones selected by 1.5μg/mL puromycin (InvivoGen, Cat# ant-pr1). Sequence of ARID1B shRNA (TRCN0000238628) used was as follows: CCGGGCCGAATTACAAACGTCATATCTCGAGATATGACGTTTGTAATTCGGCTTTTTG. The control scrambled shRNA (Sigma-Aldrich, Cat# SHC002) does not target any known mouse gene.

Antibodies and Reagents

Antibodies used are as follows: Anti-ARID1A (Cat#12354), anti-ACTIN (Cat#4970), anti-P44/42 MAPK (Cat#4695), anti-pP44/42 MAPK(Erk1/2)(Thr202/Tyr204) (Cat#4370), anti-p53 (rodent specific, Cat#32532), anti-MSH2 (Cat#2017), anti-MSH6 (Cat#3995), anti-SOX2 (Cat#14962), anti-Phospho-H2A.X(Ser139) (for WB, Cat#9718), anti-Mouse HRP-linked (Cat#7076), anti-Rabbit HRP-linked (Cat#7074) from CST; Anti-p21 (Cat#ab1019199) and anti-MLH1 (Cat#ab92312) from Abcam; Anti-Phospho-H2A.X (Ser139) (Cat#05-636) from Millipore; Anti-ARID1B (Cat#A301-046A) from Bethyl Laboratories; Anti-Claudin 18 (Cat#7000178), anti-Rabbit Alexa Fluor 488 (Cat#A21206), anti-Mouse Alexa Fluor Plus 555 (Cat#A32727) from ThermoFisher Scientific; Anti-PMS2 (Cat#556415) and PI/RNase staining solution (Cat#550825) from BD Biosciences; Anti-53BP1 (Cat#NB100-304) from Novus Biologicals. GSK126, F10058-F4, Panobinostat, ATRi (VE822), Cisplatin and Doxorubicin, were purchased from Selleckchem.

Flow Cytometric Analyses

Cell cycle profiles were measured by flow cytometry using propidium iodide (PI) as described before [4].

Western Blot Analysis

Immunoblotting was performed as described before [4]. Briefly, cellular proteins were extracted in RIPA lysis buffer (Sigma-Aldrich, Cat#R0278) plus protease and phosphatase inhibitors (Sigma-Aldrich, Cat#P0044, P5726, P8340) and separated by SDS–PAGE. Membranes with transferred protein were incubated with primary antibodies at 4 °C overnight followed by HRP-conjugated

secondary antibodies and visualized using the enhanced chemiluminescence (ECL) detection system (Biorad, Cat#1705061) by ChemiDoc (Biorad) scanning.

Quantitative RT–PCR

Total RNA was extracted using the RNeasy Mini kit (Qiagen, Cat#74106) and reverse transcription performed using High-Capacity cDNA Reverse Transcription Kit (ThermoFisher Scientific, Cat#4368813) according to the manufacturer's instructions. Quantitative RT–PCR were performed using the StepOnePlus Real-Time PCR System (ThermoFisher Scientific), with predesigned TaqMan gene expression assays (ThermoFisher Scientific): Actb (Mm00607939_s1), ARID1B (Mm01338353_m1), SNAI1 (Mm00441533_g1), SNAI2 (Mm00441531_m1), ZEB1 (Mm00495564_m1), ZEB2 (Mm00497196_m1), VIM (Mm01333430_m1), CDH2 (Mm01162497_m1), SOX2 (Mm03053810_s1), CLDN18 (Mm00517321_m1) and EZH2 (Mm00468464_m1). RT-PCR reactions were performed in triplicate and analysis done as described before [4] with data normalized on "KC" as control.

MMR Assay

The MMR assay was performed as previously described [5]. Briefly, cells were seeded in 12-well plates and were transfected with a plasmid mixture containing 160 ng of pmax-mOrange (vector control) or pmax-G:G-mismatch-mOrange (MMR) together with 840 ng of carrier DNA . After 48 hours of transfection, cells were harvested and analysed by BD FACSCELESTA flow cytometer (BD Biosciences). The relative MMR capacity, expressed as % of reporter expression (R.E.) was determined by dividing the percentage of mOrange-positive cells in MMR by the percentage of mOrange-positive cells in vector control.

RNA-Sequencing and Bioinformatic Analysis

Total RNA was isolated from cultured "KC" and "KAC" cell lines, using RNeasy Mini kit (Qiagen, Cat#74106) and integrity validated using the Agilent 2100 Bioanalyzer. Libraries were generated using 1–2μg of total RNA following the Illumina protocol for preparing samples for high throughput stranded mRNA sequencing on the Illumina NextSeq 500 High sequencer using the 76nt PE format. As described previously [6], sequence reads were aligned to the GRCm38 mouse genome with tophat (v2.0.13) with parameters allowing a read to be mapped to at most one location: "--no-coverage-search -p 1 -g 1". Gene hits were counted with HTseq (v0.6.1) under default parameters using release 84 of the Mus_musculus.GRCm38 GTF annotation file. Differential expression analysis was performed in R/Bioconductor following the DESeq2 workflow [7] and annotated with biomaRt.

ATAC-seq

A suspension of 50,000 cells from both "KC" and "KAC" cultures in cold lysis buffer was incubated in transposition reaction mixture at 37 °C and subjected to ATAC-seq, as described previously [6]. Briefly, after the reaction was immediately purified by the Qiagen MinElute PCR Purification Kit, PCR was performed on the eluted DNA using barcoded primer and thermal cycle according to the method described above. We used Bowtie 2 (version 2.2.3) with parameters allowing for soft clipping to align the sequencing reads for each sample to the NCBI reference mouse genome sequence (GRCm38). Peak calling for each sample was performed using MACS2 (version 2.1.0) with default parameters. For discovering differential peaks between samples, a new set of peak ranges was constructed, formed from the union of individual peaks, merging those within 200 bp of each other, and then enumerated by counting the reads mapped to each region. Peak region counts were normalized following the DESEQ2 workflow, annotated with biomaRt, and genes were selected by a threshold on fold change. Identification of transcription factor binding site motifs was done using HOMER with default parameters for motif identification [8]. Peaks were localized at known promoters and enhancers of genes available at ENCODE for the mouse genome, to confirm chromatin accessibility at regulatory loci.

References

1. Gao, X.; Tate, P.; Hu, P.; Tjian, R.; Skarnes, W.C.; Wang, Z. ES cell pluripotency and germ-layer formation require the SWI/SNF chromatin remodeling component BAF250a. *Proc. Natl. Acad. Sci.* **2008**, *105*, 6656–6661, doi:10.1073/pnas.0801802105.
2. Kawaguchi, Y.; Cooper, B.; Gannon, M.; Ray, M.; Macdonald, R.J.; Wright, C.V. The role of the transcriptional regulator Ptf1a in converting intestinal to pancreatic progenitors. *Nat. Genet.* **2002**, *32*, 128–134, doi:10.1038/ng959.
3. Jackson, E.L.; Willis, N.; Mercer, K.; Bronson, R.T.; Crowley, D.; Montoya, R.; Jacks, T.; Tuveson, D.A. Analysis of lung tumor initiation and progression using conditional expression of oncogenic K-ras. *Genes Dev.* **2001**, *15*, 3243–3248, doi:10.1101/gad.943001.
4. Gupta, S.; Pramanik, D.; Mukherjee, R.; Campbell, N.; Elumalai, S.; De Wilde, R.F.; Hong, S.-M.; Goggins, M.G.; De Jesus-Acosta, A.; Laheru, D.; et al. Molecular determinants of retinoic acid sensitivity in pancreatic cancer. *Clin. Cancer Res.* **2011**, *18*, 280–289, doi:10.1158/1078-0432.CCR-11-2165.
5. Nagel, Z.D.; Margulies, C.M.; Chaim, I.A.; McRee, S.K.; Mazzucato, P.; Ahmad, A.; Abo, R.P.; Butty, V.L.; Forget, A.L.; Samson, L.D. Multiplexed DNA repair assays for multiple lesions and multiple doses via transcription inhibition and transcriptional mutagenesis. *Proc. Natl. Acad. Sci.* **2014**, *111*, E1823–E1832, doi:10.1073/pnas.1401182111.
6. Bhattacharyya, S.; Pradhan, K.; Campbell, N.; Mazdo, J.; Vasantkumar, A.; Maqbool, S.; Bhagat, T.D.; Gupta, S.; Suzuki, M.; Yu, Y.; et al. Altered hydroxymethylation is seen at regulatory regions in pancreatic cancer and regulates oncogenic pathways. *Genome Res.* **2017**, *27*, 1830–1842, doi:10.1101/gr.222794.117.
7. Love, I.M.; Huber, W.; Anders, S. Moderated estimation of fold change and dispersion for RNA-seq data with DESeq2. *Genome Biol.* **2014**, *15*, 002832, doi:10.1101/002832.
8. Heinz, S.; Benner, C.; Spann, N.; Bertolino, E.; Lin, Y.C.; Laslo, P.; Cheng, J.X.; Murre, C.; Singh, H.; Glass, C.K. Simple combinations of lineage-determining transcription factors prime cis-regulatory elements required for macrophage and b cell identities. *Mol. Cell* **2010**, *38*, 576–589, doi:10.1016/j.molcel.2010.05.004.

© 2020 by the authors. Licensee MDPI, Basel, Switzerland. This article is an open access article distributed under the terms and conditions of the Creative Commons Attribution (CC BY) license (http://creativecommons.org/licenses/by/4.0/).
